# Clinical features of and risk factors for normoalbuminuric diabetic kidney disease in hospitalized patients with type 2 diabetes mellitus: a retrospective cross-sectional study

**DOI:** 10.1186/s12902-021-00769-8

**Published:** 2021-05-22

**Authors:** Qi Dai, Nan Chen, Ling Zeng, Xin-Jie Lin, Feng-Xiu Jiang, Xiong-Jie Zhuang, Ze-Yuan Lu

**Affiliations:** Department of Endocrinology, The Eighth Affiliated Hospital, Sun Yat-sen University, Shenzhen, 518033 China

**Keywords:** Type 2 diabetes mellitus, Normoalbuminuric diabetic kidney disease, Macrovascular complications, Risk factors

## Abstract

**Background:**

Normoalbuminuric diabetic kidney disease (NADKD) is a newly defined DKD, the clinical features and pathogenesis for which are still being understood. This study aimed to investigate the features and risk factors for NADKD in patients with type 2 diabetes mellitus (T2DM).

**Methods:**

A retrospective cross-sectional study was conducted. The related clinical and laboratory data of patients with T2DM hospitalized between August 2012 and January 2020 were collected for statistical analysis. We classified the patients with T2DM into four groups on the basis of the presence or absence of albuminuria and reduced estimated glomerular filtration rate (eGFR). Analysis of variance, the Kruskal–Wallis test, and the chi-square test were used to compare the groups. Binary logistic regression analyses with a forward stepwise method were performed to explore the risk factors for renal dysfunction in hospitalized patients with normoalbuminuric T2DM.

**Results:**

Among the 1620 patients evaluated, 500 (30.9%) had DKD, of which 9% had NADKD. The prevalence of stroke, cardiovascular events, carotid plaque, and peripheral arterial disease in NADKD was significantly higher than in a non-DKD control group (normoalbuminuric T2DM patients with eGFR of ≥60 ml/min/1.73 m^2^). Regression analyses revealed that three significant independent factors were associated with NADKD: age (OR = 1.089, confidence interval [CI] 95% [1.055–1.123], *p* < 0.001), previous use of renin−angiotensin system inhibitors (RASIs; OR = 2.330, CI 95% [1.212–4.481], *p* = 0.011), and glycated hemoglobin (HbA1c; OR = 0.839, CI 95% [0.716–0.983], *p* = 0.03).

**Conclusions:**

NADKD is mainly associated with macrovascular rather than microvascular complications. NADKD is more common in patients with normoalbuminuric T2DM with older age, previous use of RASIs, and good glycemic control.

## Background

Being the most common microvascular complication of diabetes mellitus (DM), diabetic kidney disease (DKD) is presently the leading cause of end-stage renal disease [1]. In the past, the main indicator for the diagnosis of and treatment for DKD was albuminuria, which was quantified by urinary albumin-to-creatinine ratio (UACR); however, this belief has been challenged recently [2]. In 2019, the American Diabetes Association (ADA) stated that a clinical diagnosis of DKD is usually made on the basis of the presence of albuminuria and/or reduced estimated glomerular filtration rate (eGFR) in the absence of signs or symptoms of other primary causes of kidney damage [3], which is consistent with the 2019 Chinese clinical practice guidelines for DKD [4]. According to the diagnostic criteria, normoalbuminuric DKD (NADKD) is an emerging type of DKD and features a normal UACR and reduced eGFR.

The prevalence of NADKD—in which the prevalence of albuminuria declines while the prevalence of eGFR increases—has significantly increased in recent decades [5, 6]. In China, NADKD accounted for 47.6% of DKD in outpatients and 7.0% in inpatients, meaning that NADKD has not attracted sufficient attention in clinical work [7, 8]. Before the diagnostic criteria for DKD were determined, An et al. proposed that NADKD is an early renal complication for those with a shorter duration of diabetes and a lower prevalence of diabetic retinopathy (DR) [9]. Mottl et al. showed that NADKD is more common in women than men and in patients with diabetes who have well-controlled blood pressure (BP) and glycemia [10]. Recently, Du et al. suggested that type 2 diabetes mellitus (T2DM) patients with NADKD presented with an older age and well-controlled glycemia but had poorly controlled BP and serum lipids, whereas Gong et al. showed that NADKD was more common in younger patients with T2DM who had a shorter diabetes duration, higher body mass index (BMI), and better controlled hypertension and hyperlipidemia [7, 8]. The clinical features and pathogenesis of NADKD remain controversial; however, some conclusions are no longer applicable after updating the guidelines and determining the DKD diagnostic criteria. This study aimed to explore the clinical features and risk factors of NADKD in patients with T2DM and provides a clinical research basis for further studies on its pathogenesis and treatment.

## Methods

### Study population

**Fig. 1:**
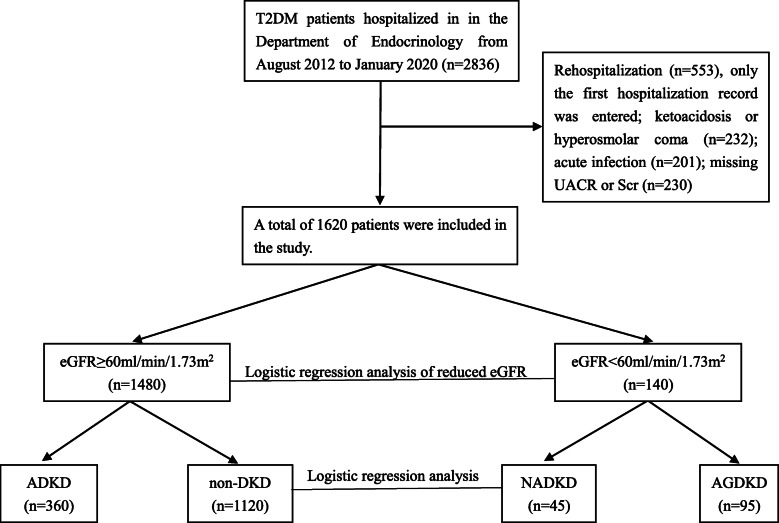
Flow diagram for an overview of the study population. T2DM type 2 diabetes mellitus, UACR urinary albumin-to-creatinine ratio, Scr serum creatinine, eGFR estimated glomerular filtration rate, non-DKD: normal control group: (UACR< 30 mg/g and eGFR≥60 ml/min/1.73m^2^), ADKD: ADKD with albuminuria but without reduced eGFR (UACR≥30 mg/g and eGFR≥60 ml/min/1.73m^2^), NADKD: DKD with normoalbuminuria and reduced eGFR (UACR< 30 mg/g and eGFR< 60 ml/min/1.73m^2^), AGDKD: patients with albuminuria and reduced eGFR (UACR≥30 mg/g and eGFR< 60 ml/min/1.73m^2^)

A retrospective cross-sectional study was designed and implemented in patients with T2DM who were hospitalized in the Department of Endocrinology at the Eighth Affiliated Hospital of Sun Yat-sen University between August 2012 and January 2020. Inpatients with available details of serum creatinine (Scr) and UACR were enrolled in this study. For patients who met all selection criteria and had multiple hospitalization records, only the first hospitalization record was entered. Exclusion criteria were as follows: (1) patients with type 1 diabetes mellitus; (2) patients who had been rehospitalized; (3) patients with ketoacidosis, hyperosmolar coma, acute infection, pregnancy, acute cardiovascular and cerebrovascular diseases, and other stressors; and (4) patients with other types of nephropathy such as primary nephrotic syndrome and hypertensive nephropathy. A total of 1620 patients were ultimately enrolled in this study (Fig. 1). This study was examined and approved by the Ethics Committee of the Eighth Affiliated Hospital of Sun Yat-Sen University, and written informed consent was obtained from all participants.

### Clinical data collection

We collected the following clinical data through face-to-face interviews: age, sex, smoking status, use of insulin, previous use of RASIs, previous treatments with lipid-lowering medication, histories of hypertension and diabetes, and adverse cardiovascular events.

Next, we physically examined all participants; height and weight, waist and hip circumferences, and BP were assessed. BMI was calculated as weight (kg) divided by height (m) squared (kg/m^2^) [11]. The difference between systolic BP (SBP) and diastolic BP (DBP) was pulse pressure (PP) [12].

Finally, imaging and laboratory test results were collected. Blood samples were obtained from patients after overnight fasting. Fasting plasma glucose (FPG), Scr, blood urea nitrogen, total cholesterol (TC), triglycerides (TG), high-density lipoprotein cholesterol (HDL-C), low-density lipoprotein cholesterol (LDL-C), apolipoprotein A (ApoAI), apolipoprotein B (ApoB), and serum uric acid (SUA) were directly measured using colorimetric and turbidimetric assays with the Beckman AU 5800 analyzer (Beckman Coulter, Shenzhen, China). Glycated hemoglobin (HbA1c) levels were examined using a fully automated glycosylated hemoglobin analyzer HLC-723 G7 (Tosoh, Shenzhen, China). Fasting morning urine samples were collected for the determination of albumin (UAlb) and creatinine (Ucr) using an A15 Automatic Biochemistry Analyzer (Biosystems, Shenzhen, China), and urinary albumin-to-creatinine ratio (UACR) was calculated as UAlb (mg) divided by Ucr (g). Ultrasonography of bilateral carotid and bilateral lower-extremity arteries were performed with a Sequoia 512 (Acuson, Shenzhen, China) for the evaluation of plaque and stenotic severity. We calculated the eGFR using the Chronic Kidney Disease Epidemiology Collaboration’s formula recommended in the ADA [3].

### Diagnostic criteria

T2DM was diagnosed according to the 1999 World Health Organization diagnostic criteria [13]. DKD was defined as diabetes with UACR of ≥30 mg/g and/or eGFR of < 60 ml/min/1.73 m^2^ in the absence of signs or symptoms of other primary causes of kidney damage according to the ADA guidelines and Chinese clinical practice guidelines [3, 4]. DR was diagnosed by ophthalmologists using standardized fundoscopic examination according to the International Clinical Diabetic Retinopathy Disease Severity Scale [14]. Diabetic peripheral neuropathy (DPN) was evaluated by electromyography. Hypertension was defined as taking medication for hypertension or SBP of ≥140 mmHg and/or a DBP of ≥90 mmHg according to the 2010 Chinese Guidelines for the Management of Hypertension [15]. The carotid plaque score was evaluated according to a semiquantitative scale score that included the following: one site with plaques having thickness of < 2 mm (score = 1), two sites with plaques having both thickness of ≤2 mm or one site with plaques having thickness of > 2 mm (score = 2), two sites with plaques including at least one plaque with thickness of > 2 mm (score = 3), and two sites with plaques having both thickness of > 2 mm or annular plaque (score = 4), with the final score being the sum of bilateral carotid plaque scores [16]. Peripheral arterial disease (PAD) was defined as ≥50% luminal narrowing of a lower-extremity artery [17]. Anemia was defined as the adult men with hemoglobin (Hb) level of <120  g/L and adult women with Hb of < 110 g/L according to the Chinese criteria [18]. Smoking history was defined by the World Health Organization (WHO) in 1997 as those who had smoked for at least 6 months or had smoked at least 100 cigarettes in their lifetime.

Reduced eGFR was defined as eGFR of < 60 ml/min/1.73 m^2^; albuminuria was defined as UACR of ≥30 mg/g; and normoalbuminuria was defined as UACR of < 30 mg/g [3]. We classified the T2DM patients into four groups on the basis of the presence or absence of albuminuria and reduced eGFR:

- *Control* group: non-DKD (UACR < 30 mg/g and eGFR ≥60 ml/min/1.73 m^2^);
- ADKD *group*: DKD with albuminuria but without reduced eGFR (UACR ≥30 mg/g and eGFR ≥60 ml/min/1.73 m^2^);
- AGDKD *group*: DKD with albuminuria and reduced eGFR (UACR ≥30 mg/g and eGFR < 60 ml/min/1.73 m^2^); and
- NADKD *group*: DKD with normoalbuminuria and reduced eGFR (UACR < 30 mg/g and eGFR < 60 ml/min/1.73 m^2^).

### Statistical analysis

All statistical analyses were conducted using SPSS version 26.0. Continuous data with normal distribution are expressed as mean ± standard deviation (x̄ ± s), whereas non-normally distributed data are presented as median and interquartile interval [M(QL, QU)]. Count data are expressed as n (%), and comparisons among groups were performed using Pearson’s chi-square test. One-way analysis of variance with least significant difference was applied for intergroup comparisons when the data showed normal distribution and homogeneity of variance. The Kruskal−Wallis test was used to compare variables with skewed distribution. Variates with a *p*-value of < 0.1 in the univariate analysis and the risk factors reported in prior literature were included in the binary logistic regression analyses with forward stepwise method to define independent risk factors of NADKD. Significance level was set at *p* < 0.05, and confidence interval (CI) was 95%.

## Results

### General clinical features and prevalence of diabetic complications

**Fig. 2:**
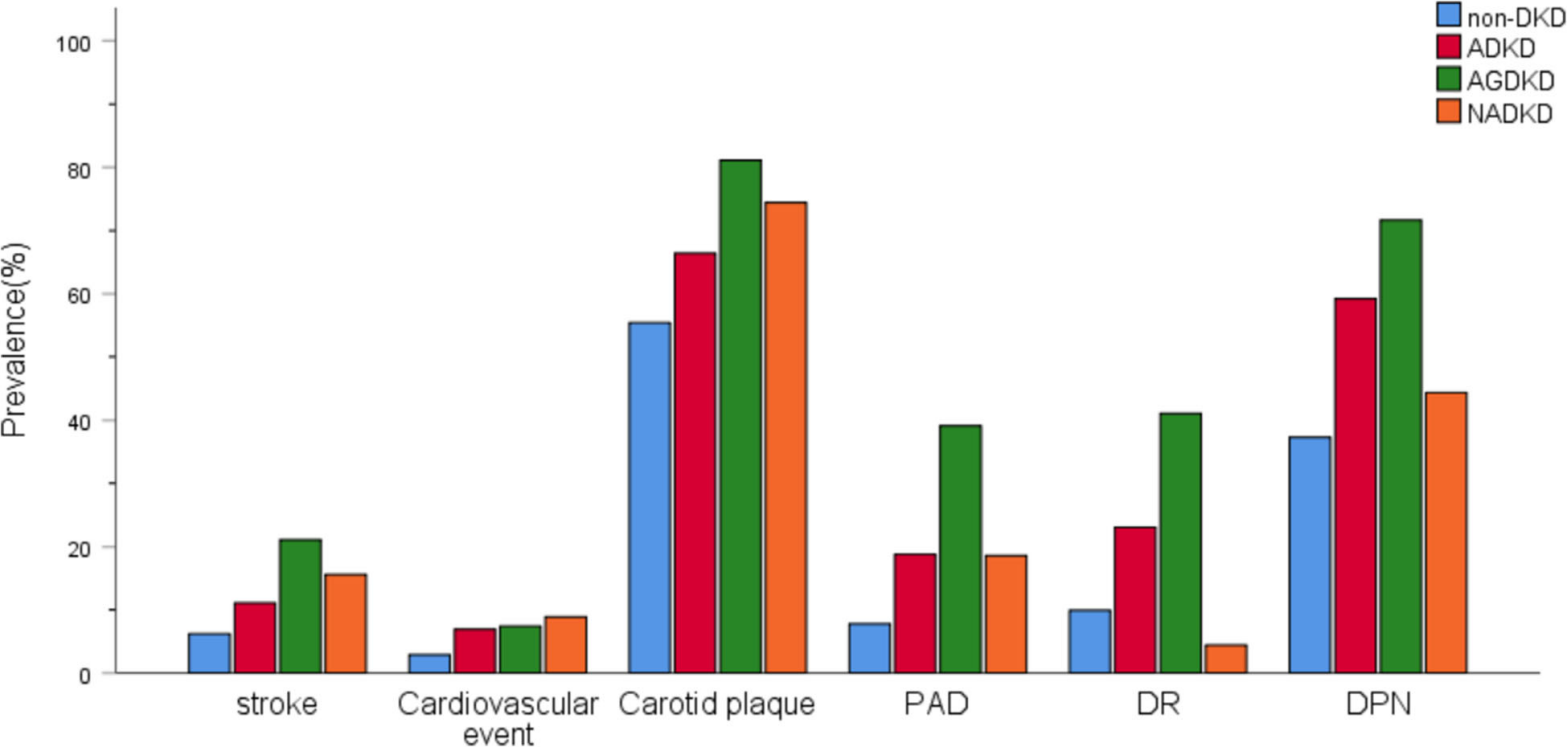
Prevalence of diabetic complications in four groups. PAD peripheral arterial disease, DR diabetic retinopathy, DPN diabetic peripheral neuropathy

Of the 1620 eligible participants, 500 (30.9%) had DKD and 45 (2.8%) had NADKD, which accounted for 9.0% of all DKD. The characteristics of the participants are presented in Table 1. The average age of all participants was 57.0 ± 12.4 years, with 34.4% of them being female. In patients with normoalbuminuric T2DM, compared with the control group (non-DKD), NADKD was significantly associated with older age; female sex; higher prevalence and longer duration of diagnosed hypertension; higher PP, higher SUA; higher use of insulin before hospital admission; higher prevalence of anemia; higher prior use of antihypertensive drugs and lipid-lowering drugs; higher prior use of RASIs; and lower levels of DBP, HbA1c, TC, ApoB, and ApoB/ApoAI. In addition, patients with NADKD had a higher prevalence of macrovascular complications than did the control group, which was mainly manifested as a higher prevalence of stroke, adverse cardiovascular event, carotid plaque, and PAD. However, there were no significant differences in the prevalence of DR and DPN. The prevalence of diabetic complications in the four groups is presented in Fig. 2. Among patients with T2DM who had a reduced eGFR, those with NADKD had a shorter duration of diabetes and a lower level of SBP, DBP, PP, TC, ApoB, ApoB/ApoAI, and prevalence of anemia than patients with AGDKD (Table 1). Furthermore, patients with NADKD had a lower prevalence of PAD, DR, and DPN, except for the prevalence of stroke, adverse cardiovascular event, and carotid plaque (Fig. 2).

**Table 1:** Basic characteristics of the participants stratified by albuminuria and reduced eGFR

Variables	non-DKD	ADKD	AGDKD	NADKD	*P*-value
Number, n (%)	1120 (69.1)	360 (22.2)	95 (5.9)	45 (2.8)	–
Age (years)	55.5 ± 11.6	57.9 ± 13.2^a^	66.2 ± 12.1^ab^	67.6 ± 12.2^ab^	< 0.001
Female	369 (32.9)	127 (35.3)	40 (42.1)	22 (48.9) ^ab^	< 0.05
Duration of diabetes (years)	6 (1, 10)	8 (3, 13) ^a^	13 (10, 20) ^ab^	8 (2, 15) ^c^	< 0.001
BMI (kg/m^2^)	24.4 (22.5, 26.5)	24.8 (22.4, 27.3)	24.2 (22.3, 25.7)	25.4 (23.2, 26.8)	0.100
WC (cm)	90.7 ± 9.1	92.1 ± 10.3	91.4 ± 9.6	92.6 ± 7.6	0.079
Smoking, n (%)	381 (34.0)	129 (35.8)	28 (29.5)	9 (20.0)	0.150
Hypertension	481 (42.9)	257 (71.4) ^a^	84 (88.4) ^ab^	34 (75.6) ^a^	< 0.001
SBP (mmHg)	135 ± 18	145 ± 21^a^	152 ± 22^ab^	137 ± 20^bc^	< 0.001
DBP (mmHg)	83 ± 11	86 ± 12 ^a^	85 ± 12	79 ± 12^abc^	< 0.001
PP (mmHg)	50 (41, 60)	56 (45, 68) ^a^	67 (50, 79) ^ab^	57 (42, 73) ^ac^	< 0.001
Duration of hypertension (years)	0 (0, 4)	1 (0, 10) ^a^	3 (0, 10) ^ab^	5 (0, 10) ^a^	< 0.001
FPG (mmol/L)	7.73 (6.27, 9.78)	8.93 (6.67, 11.36) ^a^	7.44 (5.51, 10.80) ^b^	6.90 (6.16, 9.50) ^b^	< 0.001
HbA1c (%)	9.3 ± 2.3	9.9 ± 2.2^a^	9.0 ± 2.3^b^	8.3 ± 2.5^ab^	< 0.001
Scr (μmol/L)	66 (54, 76)	70 (57, 82) ^a^	140 (114, 184) ^ab^	114 (98, 125) ^ab^	< 0.001
BUN (mmol/L)	4.85 (4.08, 5.92)	5.40 (4.40, 6.40) ^a^	8.60 (7.18, 11.05) ^ab^	6.92 (5.95, 8.95) ^ab^	< 0.001
SUA (μmol/L)	338 (280, 404)	345 (289, 418) ^a^	409 (378, 482) ^ab^	428 (332, 486) ^ab^	< 0.001
eGFR (ml/min/1.73m^2^)	100.8 (91.2, 109.2)	94.6 (78.6, 107.5) ^a^	43.0 (28.4, 49.9) ^ab^	50.7 (45.9, 56.2) ^ab^	< 0.001
UACR (mg/g)	7 (4, 12)	95 (46, 217) ^a^	432 (114, 1083) ^ab^	6 (4, 15) ^bc^	< 0.001
TG (mmol/L)	1.56 (1.04, 2.25)	1.74 (1.17, 2.78) ^a^	1.74 (1.23, 2.52)	1.34 (1.03, 1.89) ^b^	< 0.001
TC (mmol/L)	4.86 (4.08, 5.72)	5.11 (4.31, 6.18) ^a^	4.99 (4.23, 5.97)	4.38 (3.69, 5.44) ^abc^	< 0.001
HDL-C (mmol/L)	1.07 (0.92, 1.24)	1.05 (0.90, 1.28)	1.08 (0.88, 1.31)	1.10 (0.98, 1.34)	0.735
LDL-C (mmol/L)	3.02 (2.39, 3.60)	3.05 (2.40, 3.78)	2.80 (2.30, 3.77)	2.74 (1.98, 3.24)	0.221
ApoAI (mg/dL)	119.5 (107.1, 134.0)	120.0 (105.9, 138.3)	117.0 (102.4, 136.0)	125.2 (109.9, 142.5)	0.278
ApoB (mg/dL)	91.0 (74.1, 108.5)	96.8 (74.4, 114.8) ^a^	89.4 (77.3, 112.0)	79.0 (61.7, 100.3) ^abc^	< 0.01
ApoB/ApoAI	0.76 (0.60, 0.92)	0.79 (0.61, 1.01)	0.78 (0.61, 0.96)	0.65 (0.48, 0.83) ^abc^	< 0.05
Anemia, n (%)	32 (2.9)	29 (8.1) ^a^	39 (41.5) ^ab^	11 (24.4) ^abc^	< 0.001
Stroke, n (%)	69 (6.2)	40 (11.1) ^a^	20 (21.1) ^ab^	7 (15.6) ^a^	< 0.001
Adverse cardiovascular event, n (%)	32 (2.9)	25 (6.9) ^a^	7 (7.4) ^a^	4 (8.9) ^a^	< 0.01
Carotid plaque, n (%)	612 (55.4)	227 (66.4) ^a^	73 (81.1) ^ab^	32 (74.4) ^a^	< 0.001
Carotid plaque score	1 (0, 3)	2 (0, 4) ^a^	3 (1, 5) ^ab^	2 (0, 4) ^a^	< 0.001
PAD, n (%)	86 (7.8)	67 (18.8) ^a^	36 (39.1) ^ab^	8 (18.6) ^ac^	< 0.001
DR, n (%)	111 (9.9)	83 (23.1) ^a^	39 (41.1) ^ab^	2 (4.4) ^bc^	< 0.001
DPN, n (%)	418 (37.3)	213 (59.2) ^a^	68 (71.6) ^ab^	20 (44.4) ^c^	< 0.001
Insulin administration, n (%)	231 (20.6)	104 (28.9) ^a^	50 (52.6) ^ab^	17 (37.8) ^a^	< 0.001
Lipid-lowering drugs, n (%)	307 (27.4)	103 (28.6)	36 (37.9)	20 (44.4) ^a^	< 0.05
Antihypertensive drugs, n (%)	356 (31.8)	181 (50.3) ^a^	70 (73.7) ^ab^	29 (64.4) ^a^	< 0.001
RASIs, n (%)	197 (17.6)	105 (29.2) ^a^	46 (48.4) ^ab^	20 (44.4) ^ab^	< 0.001

One-way analysis of variance (ANOVA) with LSD was applied for the intergroup comparisons when the data accorded with normal distribution and homogeneity of variance. The Kruskal-Wallis test was used to compare variables with skewed distribution. Chi-Square test was applied to determine *p*-values for categorical variables. ^a^ *p* < 0.05 compared to the non-DKD group. ^b^ *p* < 0.05 compared to the ADKD group. ^c^ *p* < 0.05 compared to the AGDKD group. *BMI* Body mass index, *WC* Waist circumference, *SBP* Systolic blood pressure, *DBP* Diastolic blood pressure, *PP* Pulse pressure, *FPG* Fasting plasma glucose, *HbA1c* Glycated hemoglobin, *Scr* Serum creatinine, *BUN* Blood urea nitrogen, *SUA* Serum uric acid, *eGFR* Estimated glomerular filtration rate, *UACR* Urinary albumin-to-creatinine ratio, *TG* Triglycerides, *TC* Total cholesterol, *HDL-C* High-density lipoprotein cholesterol, *LDL-C* Low-density lipoprotein cholesterol, *ApoAI* Apolipoprotein A, *ApoB* Apolipoprotein B, *PAD* Peripheral arterial disease, *DR* Diabetic retinopathy, *DPN* Diabetic peripheral neuropathy, *RASIs* Renin-angiotensin system inhibitors

### Logistic regression analysis of NADKD and non-DKD groups

Normoalbuminuric T2DM patients were selected as the study population. Considering reduced eGFR as the dependent variable, the potential risk factors of univariate logistic regression analysis and previous research reports were entered into binary logistic regression analyses with forward stepwise method to define independent risk factors of NADKD. Ultimately, sex and age; duration of diabetes; BMI; hypertension; previous use of antihypertensive drugs, RASIs, and lipid-lowering drugs; smoking; PP; duration of hypertension; and FPG, HbA1c, TG, TC, HDL-C, LDL-C, and ApoB/ApoAI levels were considered as independent variables. Table 2 showed that age (95% CI: 1.055–1.123, *p* < 0.001), HbA1c level (95% CI: 0.716–0.983, *p* = 0.03), and previous use of RASIs (95% CI: 1.212–4.481, *p* = 0.011) were the significant factors that independently predicted NADKD.

**Table 2:** Logistic regression analysis of NADKD and non-DKD groups

Variables	β	SE	Wald	*P*	OR (95% CI)
Age	0.085	0.016	28.616	< 0.001	1.089 (1.055 ~ 1.123)
HbA1c	−0.175	0.081	4.723	0.03	0.839 (0.716 ~ 0.983)
RASIs	0.846	0.334	6.432	0.011	2.330 (1.212 ~ 4.481)

*HbA1c* Glycated hemoglobin, *RASIs* Renin-angiotensin system inhibitors

### Logistic regression analysis of reduced eGFR

All T2DM patients were selected as the study population. Considering reduced eGFR as the dependent variable, binary logistic regression analyses using forward stepwise method were performed. Results demonstrated that independent risk factors associated with reduced eGFR were age (95% CI: 1.041–1.081, *p* < 0.001), duration of diabetes (95% CI: 1.011–1.065, *p* < 0.01), hypertension (95% CI: 1.656–4.573, *p* < 0.001), and UACR (95% CI: 1.001–1.002, *p* < 0.001; Table 3).

**Table 3:** Logistic regression analysis of reduced eGFR

Variables	β	SE	Wald	*P*	OR (95% CI)
Age	0.059	0.010	37.728	< 0.001	1.061 (1.041 ~ 1.081)
Duration of diabetes	0.037	0.013	7.505	0.006	1.037 (1.011 ~ 1.065)
Hypertension	1.012	0.259	15.262	< 0.001	2.752 (1.656 ~ 4.573)
UACR	0.001	< 0.001	36.730	< 0.001	1.001 (1.001 ~ 1.002)

*UACR* Urinary albumin-to-creatinine ratio

## Discussion

This study revealed that the occurrence of NADKD is closely related to previous use of RASIs, and NADKD is more common in older patients with good glycemic control. RASIs can significantly reduce albuminuria while possibly leading to an acute decrease in eGFR, resulting in the conversion of some non-DKD patients into those with NADKD [19–21]. A total of 44.4% patients with NADKD were previously treated with RASIs in our study. This study shows that the prevalence of hypertension in patients with NADKD was significantly higher than in patients without DKD. Furthermore, our analyses revealed that hypertension as a risk factor for reduced eGFR in patients with T2DM. Hypertension and DM responses act synergistically in promoting kidney injury, which may be mediated by increased intraglomerular pressure. The synergistic effects of hypertension and DM to promote structural kidney injury may be mediated by increased intraglomerular pressure and flow, which ultimately contributes to glomerular hyperfiltration and albuminuria [22]. Holtkamp et al. showed that an acute decline in eGFR occurs in the initial phase of treatment with losartan, and the process is an acute reversible hemodynamic change rather than a structural renal function decline [20]. Clase et al. observed that eGFR decreased to a different extent after starting a RASI blockade, and 50% of the patients with a large initial decline improved to near baseline but 14% of patients showed a decline in eGFR on subsequent evaluation [19]. Therefore, RASIs are antihypertensive drugs that dilate the efferent arteriole more than the afferent arteriole, increasing the prevalence of NADKD by reducing intraglomerular pressure and flow.

We found no differences in SBP between the NADKD non-DKD groups after antihypertensive drug treatment in our study. However, DBP in the NADKD group was significantly lower than that in the non-DKD group. Consequently, the PP in the NADKD group was significantly higher than that in the non-DKD group. Several studies have reported that intensive control of BP did not universally succeed in reducing the slope of eGFR decline, and a significant increase in PP may lead to increased diastolic load and decreased renal perfusion pressure [23, 24]. Researchers have found that PP is significantly correlated with ankle brachial index and pulse wave velocity, and it has been used to indicate atherosclerosis and renal functional decline [12, 25]. Consequently, the PP and eGFR changes in patients with hypertension who were treated with antihypertensive drugs (particularly RASIs) should attract sufficient attention.

The age of patients in the NADKD group was significantly higher than that of those in the non-DKD group in our study; however, the difference in age between patients in the NADKD and AGDKD groups was not significant. In addition to hypertension, independent risk factors for reduced eGFR in patients with T2DM also include increasing age, UACR, and duration of diabetes. Age is a key determinant of prognosis in CKD [26]. O’Hare et al. pointed out that eGFR decreased with advancing age, and older patients had higher mortality rates than young patients when eGFR levels at baseline were comparable [27]. Murussi et al. speculated that the decrease in eGFR with advancing age is caused by the accumulation of mesangial matrix matter, and their study found that patients with microalbuminuria showed a higher eGFR fall rate than those with persisting normoalbuminuria [28]. Our results also showed that the decrease in eGFR was negatively associated with age and UACR.

Interestingly, HbA1c is significantly lower in the NADKD group (8.3 ± 2.5) than in the non-DKD group (9.3 ± 2.3) (*p* < 0.05) in our study. Mottl et al. has reported that NADKD is more common in patients with well-controlled glycemia [10]. Two reasons might explain this phenomenon. First, with the deterioration of renal function, the correlation between HbA1c and FPG is weakened, which is more obvious in patients with anemia [29]. HbA1c cannot accurately reflect recent blood glucose control in such situation. Anemia as a common metabolic complication of CKD can result in low HbA1c, which may be related to decreased erythropoietin synthesis and shortened red blood cell lifespan because of impaired renal function [30, 31]. Therefore, glycated albumin should be combined to determine recent blood glucose control [32, 33]. Second, the significant positive effect of HbA1c on eGFR in each stage of CKD suggests that hyperfiltration exists in all stages, and hyperglycemia induces glomerular hyperfiltration by increasing ultrafiltration coefficient and membrane permeability to low-molecular-size dextran [34, 35]. Furthermore, hyperglycemia promotes SUA excretion in states of hyperfiltration, and SUA level is inversely associated with HbA1c [36]. Although SUA could be an index of renal function, its increase may be the result of a decrease in eGFR [37, 38]. Therefore, we did not include SUA in our analyses.

Compared with patients in the non-DKD group, more patients in the NADKD group were treated with insulin before admission to our study. We found no significant differences in FPG between these groups, which suggested that HbA1c in the NADKD group decreased because of strict blood glucose control. Hyperfiltration caused by long-term hyperglycemia is relieved, which leads to reduced eGFR and elevated SUA. We believe that strict control of blood glucose is the main reason underlying lower HbA1c levels in the NADKD group than those in the non-DKD and ADKD groups.

In this study, the incidence of macrovascular complications in NADKD was significantly higher than that in the non-DKD group, whereas there was no significant difference in microvascular complications. Seo et al. suggested that carotid plaque is a significant independent predictor of renal function decline [39]. Roumeliotis et al. mentioned carotid intima-media thickness as a powerful and independent predictor of morbidity and mortality owing to DKD and a valuable tool for the stratification of patients with DKD [40]. Our study revealed that the semiquantitative scale score of carotid plaque in the NADKD group was significantly higher than that in the non-DKD group (*p* < 0.001), meaning that carotid atherosclerosis is more serious in NADKD. Wu et al. pointed out that renal function decline is an independent risk factor in the development of incident PAD in patients with a certain eGFR baseline [41]. In addition, several studies have demonstrated that the phenotype of NADKD might be primarily associated with macroangiopathy rather than microangiopathy, and NADKD can increase the risk of cardiovascular events and stroke [6, 8]. Thus, we believe that NADKD is different from previous diabetic nephropathy, which may be a new phenotype closely related to macroangiopathy.

Previous research has shown that a good correlation exists between reduced eGFR and albuminuria in some patients with DKD, whereas recent studies have revealed that the progress of albuminuria, which cannot fully reflect the impairment of renal function, is not synchronous with reduced eGFR, which might lead to DKD through a different pathogenesis [2, 42, 43]. First, during DKD pathogenesis, a proportion of patients with a rapid eGFR decline exhibited a normoalbuminuria, which might be related to ischemic renal changes caused by atherosclerosis of the intrarenal arteries [39]. Second, during the treatment course, albuminuria was controlled while eGFR showed a continuous decline in patients treated with RASIs [6], which is the main cause for the increased prevalence of NADKD in our study. Finally, patients with T2DM with increased albuminuria are associated with thickening of the basal membranes, mesangial expansion, and podocyte damage, whereas patients with NADKD more commonly have predominant interstitial and vascular changes [6, 44]. Therefore, sufficient attention should be paid to reduced eGFR in patients with T2DM after intensive hypoglycemic and antihypertensive therapy.

The strength of our study lies in the complete clinical data of the patients, including careful history taking, physical examination, laboratory findings, and imaging analysis. However, some limitations exist. First, this was a single-center, retrospective, observational study, which may have recall bias. We also could not identify a causal relationship between NADKD and macrovascular complications. Second, we used the eGFR values instead of directly measuring GFR. Finally, coronary angiography was not performed in all participants as patients had good enough renal function, and a large number of patients did not undergo head magnetic resonance imaging or computed tomography because of the associated high costs.

## Conclusions

Our study demonstrated that the high prevalence of NADKD is closely related to the widespread use of RASIs, and NADKD is primarily associated with macrovascular complications rather than microvascular complications. Additionally, NADKD is more common in patients with normoalbuminuric T2DM who are of an older age, have a previous use of RASIs, and have good glycemic control. Therefore, it is necessary to pay attention to decreased renal function in patients with T2DM after intensive hypoglycemic and antihypertensive therapy.

## Data Availability

The datasets analyzed during the current study are available from the corresponding author on reasonable request.

## Abbreviations

DM: Diabetes mellitus
DKD: Diabetic kidney disease
ESRD: End-stage renal disease
UACR: Urinary albumin-to-creatinine ratio
ADA: The American Diabetes Association
eGFR: Estimated glomerular filtration rate
NADKD: Normoalbuminuric diabetic kidney disease
RASIs: Renin-angiotensin system inhibitors
T2DM: Type 2 diabetes mellitus
T1DM: Type 1 diabetic patients
WC: Waist circumference
BMI: Body mass index
SBP: Systolic blood pressure
DBP: Diastolic blood pressure
PP: Pulse pressure
FPG: Fasting plasma glucose
Scr: Serum creatinine
BUN: Blood urea nitrogen
TC: Total cholesterol
TG: Triglycerides
HDL-C: High-density lipoprotein cholesterol
LDL-C: Low-density lipoprotein cholesterol
ApoAI: Apolipoprotein A
ApoB: Apolipoprotein B
SUA: Serum uric acid
HbA1c: Glycated hemoglobin
CKD-EPI: Chronic Kidney Disease Epidemiology Collaboration
WHO: World Health Organization
DR: Diabetic retinopathy
DPN: Diabetic peripheral neuropathy
PAD: Peripheral arterial disease
Hb: Hemoglobin
ADKD: Diabetic kidney disease with albuminuria but without reduced eGFR
AGDKD: Diabetic kidney disease with albuminuria and reduced eGFR
ANOVA: One-way analysis of variance
ABI: Ankle brachial index
PWV: Pulse wave velocity
CKD: Chronic kidney disease
EPO: Erythropoietin
RBC: Red blood cell
CIMT: Carotid intima-media thickness
